# Effects of particle size and thickness of quartz sand on the webbed foot kinematics of mallard (*Anas platyrhynchos*)

**DOI:** 10.1242/bio.060012

**Published:** 2023-09-05

**Authors:** Dianlei Han, Hairui Liu, Jinrui Hu, Qizhi Yang

**Affiliations:** School of Agricultural Engineering, Jiangsu University, Zhenjiang, Jiangsu, 212013, China

**Keywords:** Substrate, Particle, Mallard foot, Webbed foot, Joint, Locomotion strategies

## Abstract

The webbed foot structure of mallards (*Anas platyrhynchos*) exhibits effective anti-subsidence properties when walking on soft ground. To investigate the effects of quartz sand particle size and thickness on joint angles and the movement patterns of webbed feet, we created a testing substrate with quartz sand and utilized high-speed cameras and kinematic analysis tools for data acquisition. Mallards mainly adjusted the tarsometatarso-phalangeal joint (TMTPJ) during touch-down and lift-off stages in response to increasing particle size or enhanced ground roughness. Conversely, adjustments to the intertarsal joint (ITJ) predominantly took place during mid-stance. Conversely, mallards predominantly adjusted the ITJ during touch-down and lift-off when coping with increased quartz sand thickness, with TMTPJ adjustments mainly occurring at touch-down. As quartz sand particle size increased, the TMTPJ angle increased, the ITJ angle decreased, toe closure advanced, and the duty factor decreased throughout the entire stride cycle. In contrast, increasing quartz sand thickness led to more delayed TMTPJ adjustments, slower webbed foot closure, and an increased duty factor throughout the stride cycle. Mallards modify their leg posture to notably decrease the touch-down foot angle upon encountering sandy terrain. This action subsequently forms a depression beneath their feet, contributing to sand consolidation and limiting flow. During the stance phase, the mallard's weight is distributed across the webbed foot, generating minimal pressure and preventing significant subsidence while walking on sandy ground.

## Teaser

**Summary:** Effects of particle size and thickness of quartz sand on the joint (tarsometatarso-phalangeal joint and intertarsal joint) angle and the webbed foot kinematics of mallard.

## INTRODUCTION

AnimalTable S1 limbs and feet, functioning as actuators in direct contact with the ground during locomotion on soft terrain, must perform several crucial tasks to meet the biomechanical requirements of movement. These tasks include vibration dampening, sand fixation and flow restriction, adhesion, sand crossing, and energy conservation. Limbs and feet primarily comprise bones, muscles, tendons, joints, and soft tissues. Different toe structures and components play distinct roles in animal locomotion. For instance, plantar grooves assist in sand retention, limiting its flow, while plantar features such as the mastoid process, bristles, and veins contribute to enhanced ground contact and adhesion. The integrated assembly of tendons, bones, and joints enables continuous adjustment of toe locomotion posture, thereby improving traction performance.

Mallards, as semi-aquatic birds, retain the ability to walk on land and frequently inhabit tidal flats, river bays, and similar environments. Their webbed feet readily adapt to both walking and swimming on soft terrain. Current research indicates that the coexistence of toes and webbing in mallard feet plays a crucial role in walking on sandy surfaces. However, specific adaptations in movement posture that the concurrent toes and webbing make to counteract sinking on soft ground have yet to be investigated. Additionally, the role of webbed feet compared to non-webbed birds such as pheasants warrants further investigation.

Falkingham and Gatesy (2014) utilized equipment such as X-rays and high-speed cameras to examine the hind limb posture of guineafowl walking through poppy seeds. They employed discrete element software to simulate toe locomotion on poppy seeds and analyzed disturbances at varying depths caused by the toes. Zhang et al. (2018) applied reverse engineering technology to reconstruct the touch-down posture of an ostrich foot at five instantaneous moments. They combined discrete element software simulations of the ostrich foot's sand touch-down process with an analysis of particle micro-behavior under toe action. The study revealed that the velocity field, force field, and perturbation range of particles during running gaits are significantly larger than those observed in walking gaits. The above scholars carried out discrete element simulation on the soft medium kinematics of birds.

To explore the kinematics and inverse dynamics of reindeer front and rear hooves, Li et al. (2020) investigated the joint angle variation patterns, net joint moments, net joint power, and work at different velocities. Utilizing a motion tracking system and plantar pressure data, they conducted an in-depth analysis of hoof joint function. Both front and rear hooves in reindeer serve as elastic energy storage systems, albeit with distinct roles. In particular, the front hoof maintains stability, while the hind hoof provides propulsion. Schaller et al. (2011) investigated the function and dynamic plantar pressure distribution of the toes during locomotion on solid surfaces using plantar pressure plates and high-speed video cameras. The sole surviving third toenail acts as a rigid element, serving as a positional anchor when embedded in the substrate at higher speeds. The fourth toe plays a crucial role in maintaining balance during walking and serves as a vital load-bearing element. Zhang et al. (2017b) assessed ostrich plantar pressure during locomotion on sand and solid surfaces with the aid of plantar pressure plates. They analyzed the effects of gait and substrate on plantar pressure, finding that gait exerts a more significant influence than the substrate. The third toe of the ostrich foot functions as the primary support, providing effective traction by compacting the sand, while the fourth toe offers secondary support, ensuring continuous ground clearance of the metatarsophalangeal joint and lateral stability of the entire toe. The researchers tested plantar pressure in birds.

Li et al. (2012) examined the kinematics and mechanics of zebra-tailed lizards on solid and sandy surfaces with the aid of high-speed cameras and soil troughs. They uncovered how leg, foot, and media mechanics coordinate to enhance locomotor performance. During locomotion, toe shapes changed dynamically: ‘1’ at touchdown, ‘C’ at mid-stance, ‘1’ at lift-off, and reversed ‘C’ at mid-swing. Toe speed also varied, with slower speeds during the touchdown phase and faster speeds in the swing phase. The high traversability of zebra-tailed lizards on sandy terrain is related to toe locomotion attitude, speed, and substrate mechanics. Bergmann et al. (2017) analyzed changes in spatiotemporal parameters and joint angles in lizards by employing a high-speed camera and various substrates. Lizards adjusted to changes in substrate particle size chiefly by varying stride frequency instead of stride length. Meanwhile, the angles of forelimb and hindlimb protraction and retraction remained unchanged, irrespective of substrate type or particle size. Stride frequency was highest on medium-sized particles, where lizards moved most rapidly. Duty factors tended to decrease with increasing particle size when walking on glass beads and rocky substrates (Bergmann et al., 2017). Qian et al. (2015) investigated how locomotor performance (average forward speed) changed with ground penetration resistance and robot leg frequency using a bio-inspired hexapedal robot as a physical model. Zhang et al. (2017a, 2018) utilized high-speed camera technology and Simi-Motion software to examine the angular variations of ostrich toe joints and the shifting of metatarsophalangeal joints when moving on both solid and sandy grounds. The findings revealed that toe joint angle changes were not significantly different when ostriches walked or ran on sandy terrain. Both the third and fourth toes cooperatively function as an integrated system in ostrich locomotion. Mazouchova et al. (2010) investigated the locomotion of sea turtles (*Caretta caretta*) on solid and sandy terrains using high-speed cameras and fluidized beds. They observed that the webbed feet did not slip during locomotion on either surface, primarily due to the formation of a hardened area behind the webbed feet that restricts sliding. The kinematic characteristics of animals on sandy terrain have been determined by the aforementioned researchers.

Woodward and Sitti (2018) investigated the anti-slip mechanism of desert locusts using equipment such as high-speed cameras and platforms with variable roughness. The anti-slip function of the desert locust foot is primarily achieved through the synergistic action of foot spines and foot pads. Upon contact with a non-uniform surface, the spines on the foot enhance the angle of touchdown, thus optimizing friction and opportunities for interlocking. The foot pads can secrete an emulsion that forms numerous capillary bridges between the foot and the surface, enhancing friction through adhesive forces. Autumn et al. (2000) quantified the adhesive forces on an individual gecko seta during horizontal, vertical, or particular angled movement across a surface using a scanning electron microscope and a two-dimensional microelectromechanical cantilever beam system. The adhesive forces originated from van der Waals forces between the gecko seta and the interface, and the adhesive effect was also influenced by the gecko's toe posture. The distinct direction of loading and the application of preloading force can augment the adhesion between the setae and the interface, multiplying the observed friction force by a factor greater than 600. El-Gendy et al. (2012) employed optical microscopy to observe the distribution of papillae in various regions of the ostrich plantar. Zhang et al. (2016) examined the appearance of ostrich mastoids using gross anatomy, stereomicroscopy, and scanning electron microscopy. Zhang et al. (2021) explored the role of papillae in the interaction between ostrich feet and sand using an animal plantar attachment characteristics measurement device. They discovered that the attachment coefficient of ostrich feet with papillae was greater than that of ostrich feet without papillae, indicating that papillae play a vital and indispensable role in enhancing the attachment of ostrich feet to sand. The above researchers studied the interaction between foot pads and media of different animals and the unique microscopic structure of biological feet. But the structure of the mallards’ sole has not been studied.

Taylor-Burt and Biewener (2020) studied the hindlimb kinematics and internal contraction function of the lateral gastrocnemius muscle during take-off in water and on land for mallards. They found that ankle joint angle changes were not significant between substrates, while the hip and metatarsophalangeal joints exhibited significantly greater angle changes during land take-off compared to water take-off. The knee joint extended during land take-off and flexed during water take-off. An observed increase in muscle power during water takeoff implies that creatures like mallards must modify their muscle characteristics to adapt to different environments, owing to the varying physical properties of aquatic and terrestrial substrates.

Consequently, in this study, four adult male mallards were chosen as research subjects, and two distinct substrates (fine quartz sand and coarse quartz sand) were utilized to construct loose, soft ground with varying thicknesses (2 cm, 4 cm, and 6 cm), with solid ground serving as the control group. Simi-Motion kinematic software was employed to track and analyze the instantaneous and continuous alterations in the primary joint angles of the tarsometatarso-phalangeal joint (TMTPJ), intertarsal joint (ITJ), and the webbed foot motion pattern. This allowed for a deeper understanding of the effects of particle size, thickness, and substrate composition on the webbed foot motion patterns of mallards. By studying the kinematic parameters, the effects of mallards’ foot structure and webbing on reducing ground pressure and improving anti-subsidence ability were clarified.

## RESULTS

### Spatiotemporal parameters and touchdown foot angle

Table 1 presents the spatiotemporal parameters of the mallard under seven distinct conditions. As the thickness of fine and coarse quartz sand increased, locomotion speed tended to decrease, while the stance phase duration of the mallard increased. In contrast, the swing phase duration remained relatively constant, resulting in an elevated duty factor.

**Table 1.**
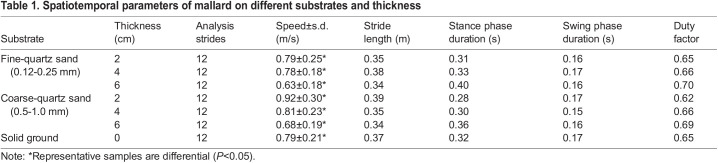
Spatiotemporal parameters of mallard on different substrates and thickness

**Fig. 1.**
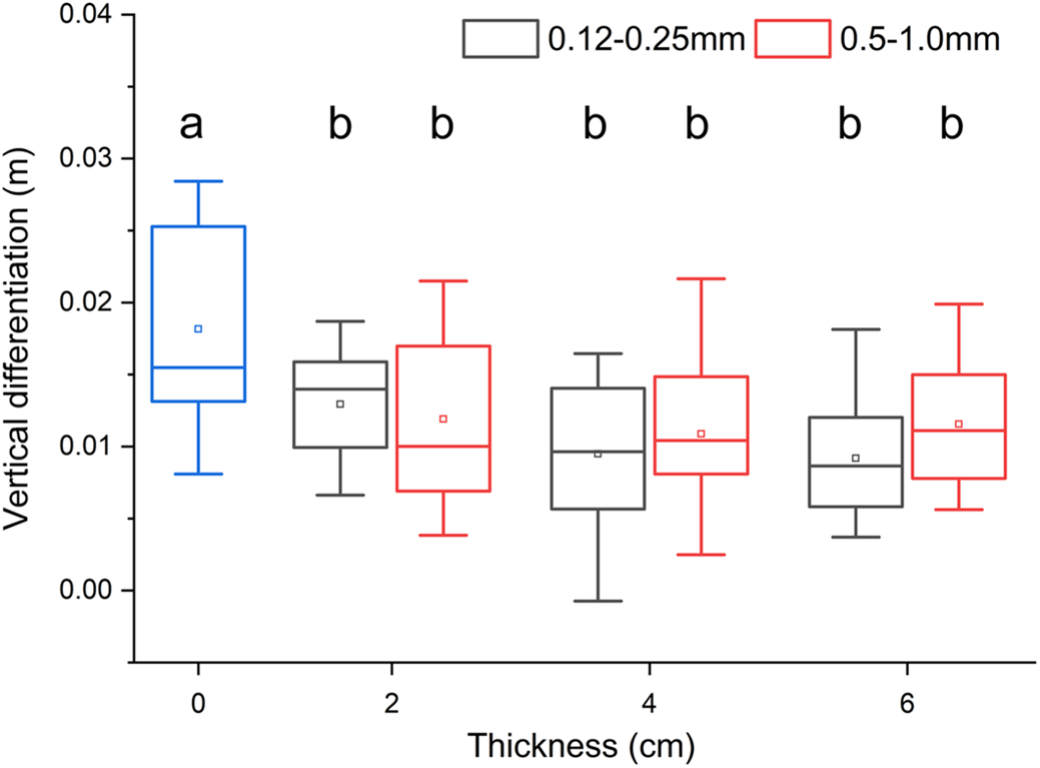
**Effect of ground softness on the touchdown angle of the mallard.** The vertical height difference values (the moment of touchdown between marker points 7 and 4 on the third toe) are shown for fine- and coarse-quartz sand at thicknesses of 0, 2, 4, and 6 cm, respectively. Twelve stride cycles were processed for each condition. Box plots display median, upper quartile, and lower quartile values, as well as maximum and minimum values, with hollow rectangles denoting mean values and solid diamonds representing outliers. Significant differences identified via *F*-test are marked by lowercase letters (*P*<0.05).

The vertical height difference between marker points 7 and 4 was greater on solid ground (quartz sand thickness of 0 cm) than on soft ground. Upon conducting a significance analysis, a notable difference was observed between solid ground and soft ground across all six conditions (Fig. 1). This finding suggests that soft ground induces a significant reduction in the touchdown foot angle, indicating that mallards adjust their leg joints accordingly before touchdown on soft ground, thereby achieving a decreased touchdown foot angle. No significant difference was observed in the mean vertical height difference among the six types of soft ground, implying that mallards adopt a nearly uniform strategy to reduce the touchdown foot angle when encountering soft ground.

### Instantaneous joint angle

**Fig. 2.**
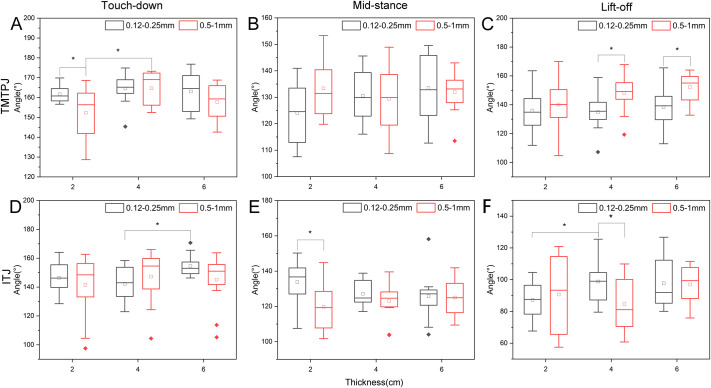
**Effect of the quartz sand particle size and thickness on the instantaneous joint angle.** Panels A-C display the joint angle variations of TMTPJ at touch-down, mid-stance, and lift-off, respectively; panels D-F exhibit the joint angle variations of ITJ at touch-down, mid-stance, and lift-off, respectively. Twelve stride cycles were processed for each condition. Box plots depict median, upper quartile, and lower quartile, as well as maximum and minimum values, with hollow rectangles signifying mean values and solid diamonds representing outliers. Significant differences identified using the *F*-test or Bonferroni test are denoted by an asterisk (*P*<0.05). TMTPJ, tarsometatarso-phalangeal joint; ITJ, intertarsal joint.

Fig. 2 illustrates the angle changes of the TMTPJ and ITJ of the mallard at touch-down, mid-stance, and lift-off. In Fig. 2A, the mean TMTPJ angle under coarse-grained quartz sand was less than that under fine-grained quartz sand, whereas in Fig. 2C, the average TMTPJ angle under coarse-grained quartz sand exceeded that under fine-grained quartz sand, in contrast to Fig. 2A. A two-way ANOVA confirmed the significance of quartz sand grain size's effect on the TMTPJ at touch-down (*P*=0.02995) and lift-off (*P*=0.00321). In Fig. 2D, the range of variation in the ITJ lift-off was greater with coarse-grained quartz sand, and a two-way ANOVA revealed that quartz sand particle size significantly impacted the ITJ at mid-stance (*P*=0.02739).

As shown in Fig. 2A, a one-way ANOVA revealed that quartz sand particle size significantly affected TMTPJ touch-down when the quartz sand thickness was 2 cm, and TMTPJ touch-down when the thickness of coarse quartz sand was between 2 and 4 cm. As displayed in Fig. 2B, neither particle size nor thickness significantly influenced TMTPJ mid-stance. As depicted in Fig. 2C, particle size significantly affected TMTPJ lift-off at quartz sand thicknesses of 4 cm and 6 cm. In Fig. 2D, fine quartz sand thickness significantly impacted ITJ touch-down at 4 cm and 6 cm. As illustrated in Fig. 2E, the average ITJ angle under coarse-grained quartz sand was smaller than that of fine-grained quartz sand, with particle size significantly affecting ITJ mid-stance at a quartz sand thickness of 2 cm. As demonstrated in Fig. 2F, particle size significantly influenced ITJ lift-off when the quartz sand thickness was 4 cm; fine quartz sand thickness significantly affected ITJ lift-off when the quartz sand thickness was 2 cm or 4 cm.

In summary, quartz sand particle size significantly affected TMTPJ at touch-down and lift-off, and ITJ at mid-stance. Increasing quartz sand particle size decreased the TMTPJ angle at touch-down and the ITJ angle at mid-stance, while increasing the TMTPJ angle at lift-off. This indicates that the mallard responded to the gradually increasing particle size or rough substrate by adjusting the TMTPJ primarily at touch-down and lift-off, with no significant postural change in the TMTPJ at mid-stance. Concurrently, the increase in particle size impacted the ITJ at mid-stance and lift-off, without significantly affecting ITJ posture at touch-down. Quartz sand thickness significantly influenced TMTPJ at touch-down and ITJ at touch-down and lift-off, with an increase in quartz sand thickness augmenting the angle. The mallard primarily adjusted the ITJ at touch-down and lift-off in response to increasing quartz sand thickness, while no significant postural change occurred in the ITJ at mid-stance. For the TMTPJ, the posture was mainly adjusted at touch-down, with no significant alterations in the TMTPJ at mid-stance or lift-off.

### Continuous joint angle

**Fig. 3.**
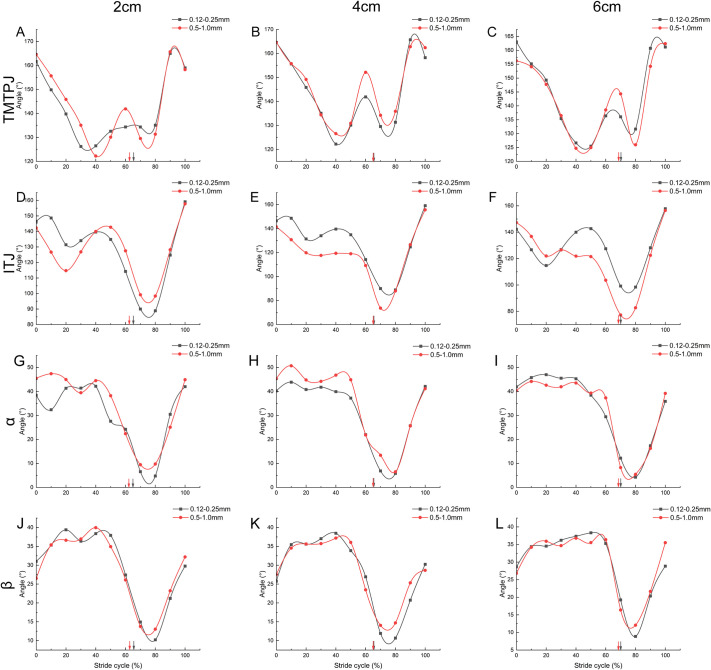
**Effect of quartz sand particle size on continuous joint angle.** A-C represent the variation patterns of quartz sand particle size effect on TMTPJ at quartz sand thicknesses of 2 cm, 4 cm, and 6 cm in a complete stride cycle, respectively; D-F represent the variation patterns of quartz sand particle size effect on ITJ at quartz sand thicknesses of 2 cm, 4 cm, and 6 cm in a complete stride cycle, respectively; G-I represent the variation patterns of quartz sand particle size effect on α joint at quartz sand thicknesses of 2 cm, 4 cm, and 6 cm in a complete stride cycle, respectively; J-L represent the variation patterns of quartz sand particle size effect on β joint at quartz sand thicknesses of 2 cm, 4 cm, and 6 cm in a complete stride cycle, respectively. Twelve stride cycles were analyzed for each condition. The arrow indicates the demarcation point between the stance and swing phase. TMTPJ, tarsometatarso-phalangeal joint; ITJ, intertarsal joint; α joint angle, between the second and third toes, β joint angle, between the third and fourth toes.

The continuous alteration pattern of TMTPJ under various substrates throughout a complete stride cycle is demonstrated in Fig. 3A-C. The overall trend of the TMTPJ angle in coarse-grained quartz sand predominantly lies above that of fine-grained quartz sand, indicating that the TMTPJ angle is smaller when the mallard contends with fine-grained quartz sand during the stance phase. The change pattern of ITJ in different substrates is illustrated in Fig. 3D-F. In contrast to the ITJ angle, the overall trend of ITJ angle in coarse-grained quartz sand is principally situated below that of fine-grained quartz sand, suggesting that the ITJ angle of the mallard is larger when dealing with fine quartz sand throughout the stance phase.

The continuous variation of α joint angle under different substrates during a complete stride cycle is presented in Fig. 3G-I, where the α joint angle maintains a maximum opening angle of 40° at touch-down and progressively decreases after leaving the ground, recovering before the subsequent touch-down. The β joint angle in various substrates is displayed in Fig. 3J-L, where the β joint angle ranges between 25-30° at touch-down and gradually increases to a maximum of 37°. As the full foot contacts the ground, the joint angle also incrementally increases to a maximum value of approximately 37°, then steadily diminishes to a minimum angle as the foot leaves the ground, returning to the initial angle before the next touch-down. The ascending curve of coarse quartz sand tends to shift to the left relative to fine quartz sand, indicating that the three toes of the mallard in the second half of the swing phase are adjusted earlier in the coarse quartz sand closure.

**Fig. 4.**
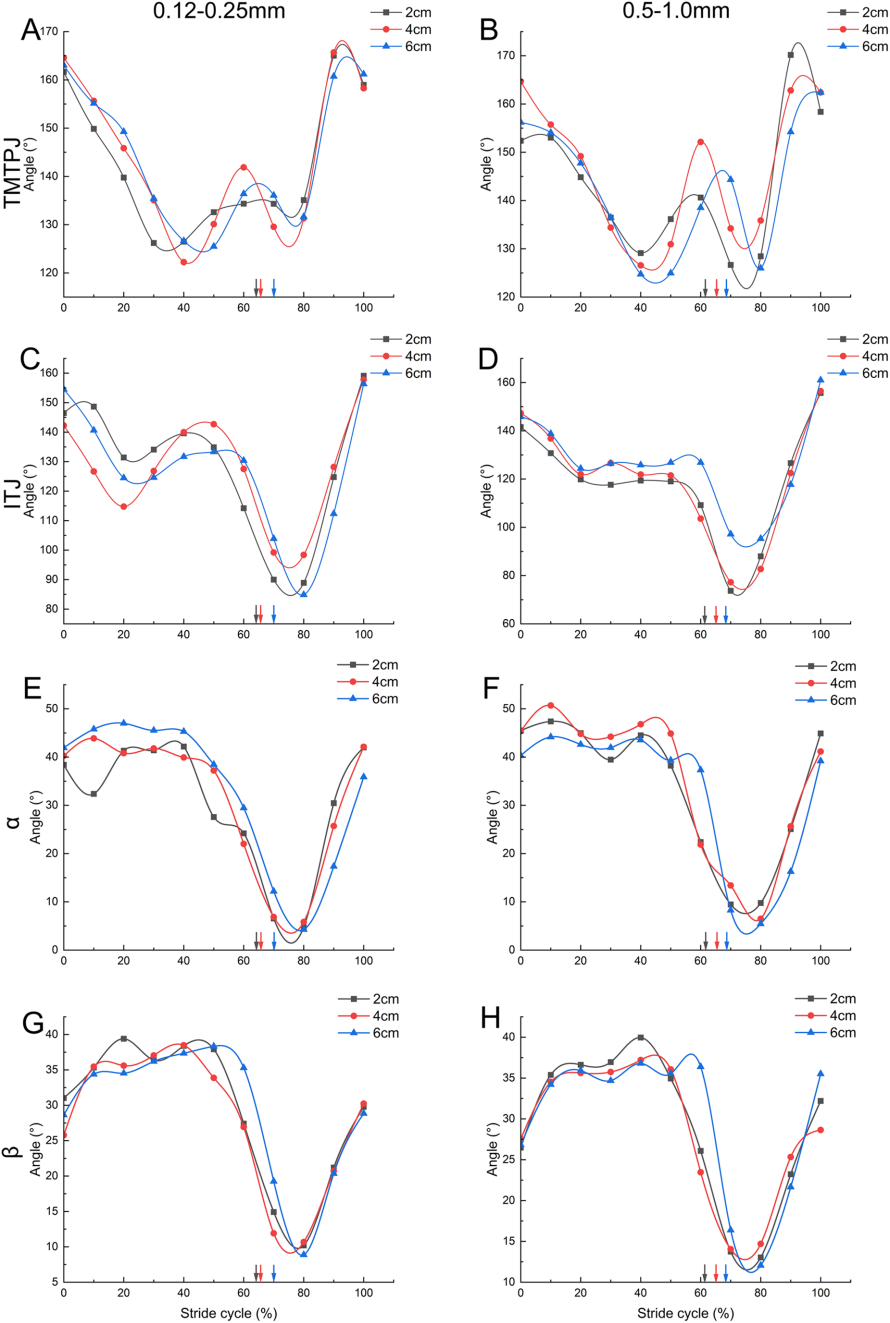
**Effect of quartz sand thickness on continuous joint angles.** A and B represent the variation patterns of the TMTPJ on fine quartz sand and coarse quartz sand with different thicknesses in a complete stride cycle, respectively; C and D represent the variation patterns of the ITJ on fine quartz sand and coarse quartz sand with different thicknesses in a complete stride cycle, respectively; E and F represent the variation patterns of the α joint on fine quartz sand and coarse quartz sand with different thicknesses in a complete stride cycle, respectively; G and H represent the variation patterns of the β joint on fine-quartz sand and coarse-quartz sand with different thicknesses in a complete stride cycle, respectively. Twelve stride cycles were analyzed for each condition. The arrow indicates the splitting point between the stance and swing phase. TMTPJ, tarsometatarso-phalangeal joint; ITJ, intertarsal joint; α joint angle, between the second and third toes, β joint angle, between the third and fourth toes.

Fig. 4A-D illustrates the impact of distinct thicknesses of coarse- and fine-quartz sand on the continuous TMTPJ angle and ITJ angle of the mallard. Fig. 4A illustrates the continuous variations of the TMTPJ angle during a full stride cycle, with the angle changes following a W-shaped pattern, ranging from 120° to 170°. The curve exhibits a slight rightward shift when the thickness increases to 4 cm and 6 cm, signifying that the elevation in quartz sand thickness delays the regulation of the mallard's TMTPJ. The ITJ angle change during the stride cycle is depicted in Fig. 4C. The ITJ angle alteration, ranging from 65° to 160°, initially decreases, then increases, followed by a rapid decline before returning to the initial value. In Fig. 4D, the ITJ angle decreases before stabilizing at a certain value, then rapidly falls and returns to its original value. This pattern suggests that the grain size might have influenced the ITJ adjustment during the stance phase. The range of variation in the ITJ angle during the stance phase was smaller than that during the swing phase.

The continuous variation of α joint angle and β joint angle at 2 cm, 4 cm, and 6 cm thicknesses of fine and coarse quartz sand during a complete stride cycle was investigated, as shown in Fig. 4E-H. The joint angle at 6 cm thickness exhibited a discernible rightward trend during the decreasing phase of the curve relative to the 2 cm and 4 cm thicknesses, indicating that a substantial increase in thickness would marginally delay the mallard's toe closure.

## DISCUSSION

### Effect of quartz sand particle size and thickness on webbed foot kinematics

When walking on soft substrates such as sand, intertidal, or muddy areas, the mallard's anti-sink function is achieved through the coordinated action of the TMTPJ and ITJ. However, at different stages of the stance phase, the mallard relies on the TMTPJ and ITJ differently. The adjustments of the TMTPJ and ITJ are subtle, with the ITJ primarily adjusted during lift-off. For beach surfaces where grain size transitions from fine to coarse, the mallard predominantly adjusts the TMTPJ at touch-down, the ITJ at mid-stance, and both the TMTPJ and ITJ in tandem at lift-off.

Regarding mallard locomotion on coarse- and fine-quartz sand, the duty factor diminishes with increasing quartz sand size (Table 1, Fig. 3) and escalates with increasing quartz sand thickness (Table 1, Fig. 4). The mallard initially contacts the ground with the tip of the third toenail, and subsequently, the foot swiftly makes contact with the ground, using the toenail as the rotation center on both solid and soft surfaces. This touchdown position ensures that the toe tips are situated on the ground while augmenting the grounded area. Nonetheless, when ambulating on solid and soft surfaces, the touchdown angles adopted differ notably. On solid surfaces, the mallard exhibits a larger touchdown angle, whereas on soft surfaces, the touchdown angle is smaller, which aligns with the strategy employed by lizards to some extent. On solid surfaces, lizards adopt a digitigrade foot posture. Throughout the stride, the hind foot solely contacts the solid surface with the toe tip. During the stance phase, the elongated toe rotates around the static toe tip and hyperextends into a ‘C’ shape. On granular surfaces, the lizard employs a plantigrade foot posture. At touch-down, the hind foot is nearly parallel to the ground, the toes are extended and remain straight, and subsequently, the foot sinks into the granular material. The touchdown foot angle is considerably smaller than that on solid surfaces (Li et al., 2012). In the 90-100% phase of the continuous change curve of the TMTPJ (Fig. 3), the curve ascends to its peak and then declines, indicating a tendency for the TMTPJ to decrease before touch-down, which was not evident in the continuous change of the ITJ. It is speculated that reducing the TMTPJ angle during locomotion on soft ground is the primary factor in reducing the mallard's touchdown angle.

### Biomechanical function of the webbed of the mallard

The three anteriorly oriented toes (second, third, and fourth toes) of the mallard are positioned horizontally for the majority of their length upon contact with a solid surface. During the later stages of the stance phase, these toes exhibit a smooth upward and forward rolling motion, lifting sequentially from the proximal to the distal end. The longest toe (third toe) rotates around the toe tip and seamlessly transitions into the swing phase without sliding backward. Mallard locomotion on soft quartz sand involves initial contact with the toenail, which remains approximately parallel to the substrate, followed by full palm contact with minimal sinking. The most notable difference from solid ground locomotion is that the toe tips pierce the substrate downward before lifting and sweeping backward and upward during withdrawal. When walking on soft ground, guineafowl immediately sink into the substrate upon foot contact, while knee height is reduced (Falkingham and Gatesy, 2014). In contrast, mallards exhibit less sinkage on soft ground due to the distribution of gravitational force over a larger webbed area throughout the stance phase, resulting in lower pressure exerted on the sandy ground. Consequently, the webbing in the toes plays a substantial role in the mallard's exceptional resistance to sinking.

**Fig. 5.**
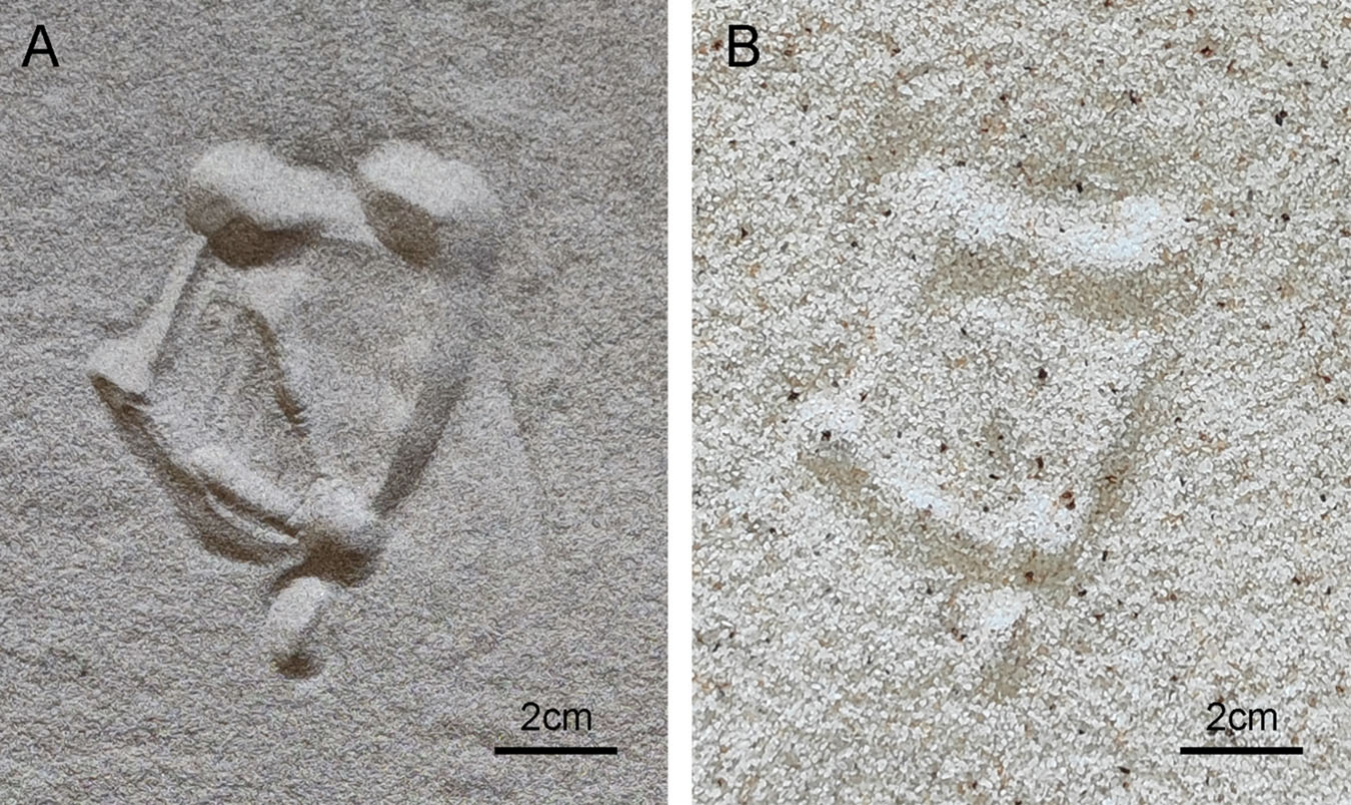
**Footprint of the right foot left by a mallard walking on fine-quartz sand (A) and coarse-quartz sand (B).** The thickness of coarse-quartz sand (particle size range 0.5-1.0 mm) and fine-quartz sand (particle size range 0.12-0.25 mm) is 4 cm.

The papillae of the ostrich foot provide stable frictional properties, and the irregular surface of its toes expands on the ground contact area (Zhang et al., 2021). The anti-slip function of the locust foot is primarily achieved through the coupling of spurs and pads (Woodward and Sitti, 2018), while gecko toes enhance adhesion via the van der Waals forces generated by the setae (Autumn et al., 2000). Mallard webbing similarly exhibits irregularly distributed rippling features on its surfaces, believed to provide sufficient friction for walking. Animal performance is sensitive to changes in ground stiffness, and having a big foot and a light body (i.e. lower foot pressure) can assist a locomotor passively minimize leg penetration ratio and remain insensitive to changes in ground stiffness (Qian et al., 2015). Newly hatched red sea turtles, capable of swimming and moving on land, utilize their webbed feet to crawl on soft sand at three times their body length per second without slipping. The high passage of the turtle is achieved by exploiting the curing properties of the granular substrate (Mazouchova et al., 2010). Mallards, as birds that can both swim and move on land, also form a hardened area under their feet (Fig. 5), which contributes to sand fixation and flow limitation. As the mallard makes contact with the ground, the toenail touches first, followed by rapid sequential contact of each toe bone segment until the entire foot is in contact. When lifting off the ground, the toes disengage from the proximal toe section by section, gradually transitioning to the toe area evenly, and the contact area progressively diminishes. This minimizes disturbance to the sandy soil and enables a smooth transition from contact posture to lift-off posture. Additionally, the mallard's center of mass shifts forward during the stance phase duration, preventing slippage on sandy ground.

The ostrich's smaller touchdown angle on loose sand generates more robust reaction forces to support its body weight. The third toe bones sequentially detach from the ground, and the supplementary force exerted by the third toe not only enlarges the contact surface but also compacts the sand beneath it. During locomotion on loose sand, the intrusion of the third toe tip into the sand is noted in the stomping phase, while the fourth toe bears a comparatively reduced load (Zhang et al., 2017a,b). The mallard's larger webbed contact area and lighter weight result in less pressure on the sandy ground, with load bearing primarily concentrated on the second, third, and fourth toes, as well as webbing. Upon ground contact, the toes initially invade the sand, and subsequent contact between the webbing and the ground restricts further toe sinking and solidifies the sand between the toes. Therefore, the structural features of the webbed foot provide a natural advantage for locomotion across sand and soft ground.

### Conclusion

An increase in quartz sand particle size results in a decrease in the TMTPJ angle at touch-down, an increase in the angle at lift-off, and a decrease in the ITJ angle at mid-stance. This leads to an augmentation in the TMTPJ angle and a reduction in the ITJ angle throughout the stride cycle in mallards, culminating in a more pronounced closure of the toes and a corresponding decline in the duty factor. Conversely, an increase in quartz sand thickness elevates the TMTPJ angle at touch-down and the ITJ angle at both touch-down and lift-off. This induces a more delayed adjustment of the TMTPJ in mallards throughout the stride cycle, resulting in a more sluggish closure of the toes and an increased duty factor. To mitigate the sinking effect of sandy terrain, mallards adjust their leg posture, significantly reducing their touchdown foot angle. Qualitatively, the initial contact of the toes with the ground involves penetration into the sand, followed by webbed contact that restricts further toe subsidence and consolidates the sand between the toes. This generates less pressure on the sandy ground, and the foot's structural characteristics provide a natural advantage for mallard locomotion across sand and loose ground. This study underscores the challenges animals face when walking on various substrates and how joint function adapts accordingly.

## MATERIALS AND METHODS

### Animal

Four 24-month-old free-ranging male mallards were selected from a specialized farm in Zhejiang Province, China, for this experiment, with a mean body weight of 1275±43.30 g (expressed as mean±s.d.). During the trial, the subjects were housed in custom-made duck enclosures and provided with ample water and food to ensure optimal living conditions. Ethical approval was granted by the Animal Experimental Ethical Inspection of Jilin University (reference No. SY202206100). Following the experiment, all mallards remained healthy and were returned to the farm.

To facilitate acclimatization during the formal trial, the mallards underwent a 2-week exercise training regimen on a sandy surface, lasting approximately 30 min, four times per week. To prevent mallards from flying away during the trial and to clearly capture the marker points with the high-speed camera, select feathers on the mallards' wings were trimmed without hindering hindlimb locomotion. Black markers were utilized to darken the joints as marker points, enabling more natural mallard toe locomotion while obtaining clear and precise video footage of the joints for efficient processing.

### Kinematic test

The test site was selected within a well-ventilated and sanitary laboratory. Coarse quartz sand (particle size range 0.5-1.0 mm) and fine quartz sand (particle size range 0.12-0.25 mm) were employed in the study. Quartz sand of varying thicknesses was manually prepared on a horizontal runway (120×50 cm, length×width), with thicknesses of 2 cm, 4 cm, and 6 cm, respectively. A solid surface served as the reference control, totaling seven experimental conditions. During quartz sand preparation, sand was evenly distributed on the horizontal surface, leveled with a brush, and thickness was measured using a straight steel rule to ensure compliance with specifications. Mallards were guided to walk at a consistent, uniform speed on the various substrates of the track using food incentives and a duck lead rope. A minimum of nine effective trials was conducted for each mallard on each substrate. The test was terminated immediately if a mallard exhibited reluctance to locomotion.

**Fig. 6.**
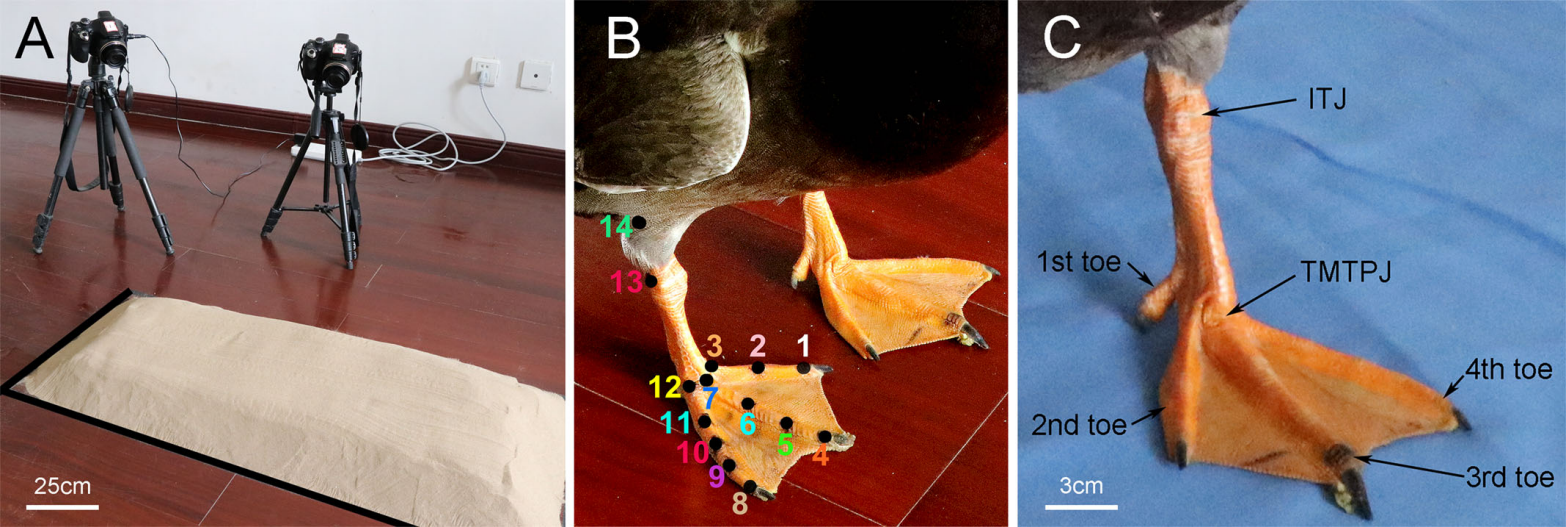
**Test environment and marker positions.** (A) Layout of the equipment (quartz sand particle size range of 0.12-0.25 mm, thickness of 6 cm). (B) Marker locations in the right foot of the mallard. (C) Joint and toe locations in the left foot of the mallard. Three-dimensional (3D) coordinates of these landmarks were used to determine the following 3D angles: the tarsometatarso-phalangeal joint angle (TMTPJ angle, 6-7-13), the intertarsal joint angle (ITJ angle, 7-13-14), the α joint angle (2-3-7-6) between the second toe and the third toe, and the β joint angle (6-7-12-11) between the third toe and the fourth toe.

A 3D capture system necessitates recording the same motion simultaneously from at least two different angles (>60°) to reconstruct the 3D spatial position. Consequently, two high-speed cameras (Casio Exilim EX-FH25, Tokyo, Japan) were utilized for 120 Hz/s video recording, positioned at the right front and right side of the sports field, respectively. It was ensured that the mallard in motion was captured within the field of view of both cameras, as depicted in Fig. 6A. A 16-point calibration frame was employed to record a calibration video from the same angle as the motion video prior to the experiment's commencement. The video recording process required maintaining a stable and unaltered camera position until both the motion video and calibration video were completed, with recalibration necessary if the camera position changed during the test.

The locomotion of the toes of the mallard was primarily completed by the second toe, the third toe and the fourth toe. Thus, the joint points on the three toes of the right foot of the mallard were marked (Fig. 6B): the second toe of the mallard has three markers in total, namely, the dorsal ridge of the toenail (marker 1), the first phalanx and the second interphalangeal joint (marker 2), and the second TMTPJ (marker 3). There were four markers in the third toe, including the dorsal ridge of the toenail (marker 4), the second phalanx and the third interphalangeal joint (marker 5), the first phalanx and the second interphalangeal joint (marker 6), as well as the third TMTPJ (marker 7). There were five markers in the fourth toe, which comprised the dorsal ridge of the toenail (marker 8), the third segment of the phalanx and the fourth segment of the interphalangeal joint (marker 9), the second segment of the phalanx and the third segment of the interphalangeal joint (marker 10), the first segment of the phalanx and the second segment of the interphalangeal joint (marker 11), as well as the fourth TMTPJ (marker 12). Furthermore, there were ITJ (marker 13) and tibiotatarsal bones (marker 14). Fig. 6C shows the location of the toe, TMTPJ and ITJ of mallard.

### Data processing

Videos of the four mallards exhibiting uniform motion speed and optimal condition were selected and imported into Simi-Motion kinematics software (Simi Reality Motion Systems GmbH, Unterschleissheim, Germany) for tracking analysis. A minimum of three complete stride cycles for each condition per mallard were processed, totaling 12 strides. Spatiotemporal parameters (stride length, stride duration, duty factor, etc.) and joint angles of the mallard's motion were obtained through 3D space recovery and calculation using Simi-Motion software. The TMTPJ and ITJ angles were analyzed at right foot touch-down (0%), mid-stance (50%), and lift-off (100%). The α joint angle between the second and third toes and the β joint angle between the third and fourth toes were defined, and the continuous variation patterns of α and β joint angles concerning quartz sand particle size and thickness were analyzed.

Exported data were compiled in Excel (Microsoft, Redmond, WA, USA), and Bessel curves in Origin Pro 2023 (OriginLab Corporation, Northampton, MA, USA) were employed to graph the continuous changes in TMTPJ, ITJ, α, and β joint angles. Box plots were utilized to graph the instantaneous changes in TMTPJ and ITJ angles. The results and patterns of the instantaneous and continuous joint angles of the mallards were analyzed. Additionally, the vertical height difference between marker points 7 and 4 on the mallard's third toe at touch-down was represented in a box plot to measure the effect of ground softness on the angle of ground contact, as the third toe touches the ground first and is approximately parallel to the forward direction.

To investigate the significant differences in quartz sand particle size and thickness on the mallard's joint angles, *F*-tests in Origin Pro were conducted, with results corrected using the Bonferroni test. One-way ANOVA tests were performed to assess the variability of each of the four mallards under each condition. The impact of quartz sand particle size and thickness on toe and web locomotion posture was evaluated by two-way ANOVA. Results were considered statistically significant if *P*<0.05.

## Supplementary Material

10.1242/biolopen.060012_sup1 Supplementary information

## References

[BIO060012C1] Autumn, K., Liang, Y. A., Hsieh, S. T., Zesch, W., Chan, W. P., Kenny, T. W., Fearing, R. and Full, R. J. (2000). Adhesive force of a single gecko foot-hair. *Nature* 405, 681-685. 10.1038/35015073 10864324

[BIO060012C2] Bergmann, P. J., Pettinelli, K. J., Crockett, M. E. and Schaper, E. G. (2017). It's just sand between the toes: How particle size and shape variation affect running performance and kinematics in a generalist lizard. *J. Exp. Biol.* 220, 3706-3716. 10.1242/jeb.161109 29046416

[BIO060012C3] El-Gendy, S. A. A., Derbalah, A. and Abu El-Magd, M. E. R. (2012). Macro-microscopic study on the toepad of ostrich (Struthio camelus). *Vet. Res. Commun.* 36, 129-138. 10.1007/s11259-012-9522-1 22382528

[BIO060012C4] Falkingham, P. L. and Gatesy, S. M. (2014). The birth of a dinosaur footprint: Subsurface 3D motion reconstruction and discrete element simulation reveal track ontogeny. *Proc. Natl. Acad. Sci. U.S.A.* 111, 18279-18284. 10.1073/pnas.1416252111 25489092 PMC4280635

[BIO060012C5] Li, C., Hsieh, S. T. and Goldman, D. I. (2012). Multi-functional foot use during running in the zebra-tailed lizard (Callisaurus draconoides). *J. Exp. Biol.* 215, 3293-3308. 10.1242/jeb.061937 22693026

[BIO060012C6] Li, G., Zhang, R., Han, D., Pang, H., Yu, G., Cao, Q., Wang, C., Kong, L., Chengjin, W., Dong, W., et al. (2020). Forelimb joints contribute to locomotor performance in reindeer (Rangifer tarandus) by maintaining stability and storing energy. *PeerJ* 8, e10278. 10.7717/peerj.10278 33240627 PMC7666566

[BIO060012C7] Mazouchova, N., Gravish, N., Savu, A. and Goldman, D. I. (2010). Utilization of granular solidification during terrestrial locomotion of hatchling sea turtles. *Biol. Lett.* 6, 398-401. 10.1098/rsbl.2009.1041 20147312 PMC2880067

[BIO060012C8] Qian, F., Zhang, T., Korff, W., Umbanhowar, P. B., Full, R. J. and Goldman, D. I. (2015). Principles of appendage design in robots and animals determining terradynamic performance on flowable ground. *Bioinspir. Biomim.* 10, 056014. 10.1088/1748-3190/10/5/056014 26448267

[BIO060012C9] Schaller, N. U., D'août, K., Villa, R., Herkner, B. and Aerts, P. (2011). Toe function and dynamic pressure distribution in ostrich locomotion. *J. Exp. Biol.* 214, 1123-1130. 10.1242/jeb.043596 21389197

[BIO060012C10] Taylor-Burt, K. R. and Biewener, A. A. (2020). Aquatic and terrestrial takeoffs require different hindlimb kinematics and muscle function in mallard ducks. *J. Exp. Biol.* 223, jeb223743. 10.1242/jeb.223743 32587070

[BIO060012C11] Woodward, M. A. and Sitti, M. (2018). Morphological intelligence counters foot slipping in the desert locust and dynamic robots. *Proc. Natl. Acad. Sci. U.S.A.* 115, E8358-E8367. 10.1073/pnas.1804239115 30135101 PMC6130395

[BIO060012C12] Zhang, R., Ma, S., Li, X., Luo, G., Xue, S. and Li, J. (2016). Macroscopic and microscopic study of integuments on ostrich (Struthio camelus) foot. *J Vet Res (Poland)* 60, 219-226. 10.1515/jvetres-2016-0032

[BIO060012C13] Zhang, R., Ji, Q., Luo, G., Xue, S., Ma, S., Li, J. and Ren, L. (2017a). Phalangeal joints kinematics during ostrich (Struthio camelus) locomotion. *PeerJ* 5, e2857. 10.7717/peerj.2857 28097064 PMC5237368

[BIO060012C14] Zhang, R., Han, D., Ma, S., Luo, G., Ji, Q., Xue, S., Yang, M. and Li, J. (2017b). Plantar pressure distribution of ostrich during locomotion on loose sand and solid ground. *PeerJ* 2017, e3613. 10.7717/peerj.3613 PMC5530993 28761792

[BIO060012C15] Zhang, R., Ji, Q., Han, D., Wan, H., Li, X., Luo, G., Xue, S., Ma, S., Yang, M. and Li, J. (2018). Phalangeal joints kinematics in ostrich (Struthio camelus) locomotion on sand. *PLoS ONE* 13, e0191986. 10.1371/journal.pone.0191986 29489844 PMC5830293

[BIO060012C16] Zhang, R., Li, G., Ma, S., Pang, H., Ren, L., Zhang, H. and Su, B. (2021). Frictional performance of ostrich (Struthio camelus) foot sole on sand in all directions. *Biomech. Model. Mechanobiol.* 20, 671-681. 10.1007/s10237-020-01409-1 33481119

