# Supplementary material for: Effects of particle size and thickness of quartz sand on the webbed foot kinematics of mallard (Anas platyrhynchos)
Source: Biol Open. 2023 Sep 5;12(9):bio060012. doi: 10.1242/bio.060012 (PMC10655869; doi:10.1242/bio.060012)
Supplement: Supplementary information [file biolopen-12-060012-s1.pdf]

Table S1. Raw data

Click here to download Table S1
